# Barriers to health service access among female migrant Ugandan sex workers in Guangzhou, China

**DOI:** 10.1186/s12939-016-0453-2

**Published:** 2016-10-14

**Authors:** Alissa Davis, Beth E. Meyerson, Blessing Aghaulor, Katherine Brown, Adisyn Watson, Kathryn E. Muessig, Ligang Yang, Joseph D. Tucker

**Affiliations:** 1 HIV Center for Clinical and Behavioral Studies, Division of Gender, Sexuality & Health, Columbia University and the New York State Psychiatric Institute, 1051 Riverside Dr, Unit 15, New York, NY 10032 USA; 2 UNC-Project China, 2 Lujing Road, Guangzhou, China; 3 Department of Applied Health Science, Indiana University School of Public Health-Bloomington, 1025 E 7th St. Rm PH116, Bloomington, IN 47405 USA; 4 Rural Center for AIDS/STD Prevention, Indiana University School of Public Health-Bloomington, Bloomington, USA; 5 School of Medicine, University of North Carolina-Chapel Hill, 321 S Columbia Street, Chapel Hill, NC 27516 USA; 6 Department of Obstetrics, Gynecology, and Reproductive Sciences, University of California-San Francisco, 505 Parnassus Avenue, Rm. 1483, San Francisco, CA 94143 USA; 7 Department of Health Behavior, Gillings School of Global Public Health, University of North Carolina-Chapel Hill, 306 Rosenau, Campus Box 7440, Chapel Hill, NC 27599 USA; 8 Guangdong Provincial STD Control Center, 2 Lujing Road, 11th Floor, Guangzhou, 510095 China

**Keywords:** Health care access, Female sex workers, Migrants, China, Uganda

## Abstract

**Background:**

Increased trade between China and Uganda has fueled trafficking of female Ugandans into China. These women may face challenges accessing health services. This study focused on examining barriers to health care access among female Ugandan sex workers in China.

**Methods:**

In 2014, we undertook in-depth interviews with 19 female Ugandan sex workers in Guangzhou, China. Interviews focused on barriers to health service access and were analyzed using an *a priori* coding framework followed by open-coding to capture emergent themes.

**Results:**

Out of 19 women, 12 women reported a history of being trafficked into China. None of the women had a valid Chinese visa. Fear of being arrested for lack of documentation discouraged women in this sample from accessing hospital services. Low pay, housing exploitation, and remittances contributed to participants’ lack of financial resources, which further inhibited their ability to access health services. Participants expressed feeling social isolation from the local community and reported mistrust of local individuals and organizations, including hospitals.

**Conclusion:**

Ugandan sex workers in China faced substantial structural barriers that limited health service access. Policy changes and the development of new programs are urgently needed to ensure these women have improved access to health services.

## Background

Migration and trade between China and African countries have increased dramatically in the past 15 years [1]. China has become Africa’s largest trading partner, exchanging about $160 billion worth of goods a year [2]. Chinese investment in Africa has seen rising numbers of Chinese migrants to the region and, in turn, rising numbers of African migrants to China [2]. Though precise estimates are difficult to obtain due to the transitory nature of the population, it is estimated that there are over 130,000 Africans in the city of Guangzhou alone [3].

Uganda, in particular, has a long history with China, dating back to 1962, when Uganda won independence from British colonial rule [4]. China was 1 of the first countries to recognize Uganda’s independence and the two countries built a relationship based on non-interference with each other’s internal affairs [4]. Now, China is involved in every sector of Uganda’s economy. Chinese entrepreneurs are importing inexpensive goods from China to Uganda [5] and China has been involved in establishing various health departments and hospitals in Uganda [6]. By 2013, bilateral trade between Uganda and China reached more than $500 million [4].

Expanded Chinese-Ugandan engagement and increased economic development has resulted in an increasing number of Ugandans migrating to China for business. Most are looking to buy cheap goods in China for import to Uganda [7]. Large numbers of African businessmen living away from their families has created a demand for African sex workers in China. Lack of adequate job opportunities for Ugandan women in their home country has led them to search for job opportunities abroad. These factors, combined with low levels of prosecution for traffickers and high profit margins, have fueled the trafficking of Ugandan women to China and coercion into sex work [8].

Though several studies have examined health care issues among male African businessmen living in China [9, 10], there is little research on the health needs of female African sex workers. Given sex workers’ increased exposure to violence and risk for untreated HIV, sexually transmitted infections (STIs) and other health conditions [11], examining barriers to health service access for this highly vulnerable population is essential in order to design effective strategies to increase their linkage to health care. We undertook qualitative research to better understand access to health services among female Ugandan sex workers in Guangzhou, China. We frame our study within the political economic context of modern China. Increased trade with poor African nations has created an environment where disadvantaged African women are trafficked to China under the guise of economic opportunity [8, 12]. A lack of legal status and legitimate employment options for African women in China forces these women to remain in the informal economy performing sex work in order to survive [13]. Moreover, race and language barriers further isolate African women from the broader society and complicate access to health care. Language is a barrier to access for most foreigners in China, but may especially contribute to unequal health care access for less educated foreigners with no knowledge of Chinese [14]. Additionally, racism and discrimination impact Africans in different ways than other foreign populations, such as Europeans, Americans, and Arabs [15]. While other foreign populations are often only viewed with curiosity, Africans have become associated with the spread of disease, drug trafficking and sex work, and are often viewed negatively by native Chinese [16, 17]. Increased levels of racism and discrimination against Africans are likely to deter female African sex workers from accessing care and prevent them from integrating into the local community. Based on this context (trafficking and legal status, economic disenfranchisement, and social isolation), we sought to examine factors that contribute to Ugandan sex workers’ unequal access to health care services in Guangzhou, China.

## Methods

### Study setting

Guangzhou is a hub of international trade and commerce located in south China. The city has about 13 million people, a large number of whom emigrated from other areas in China [18]. The city also has the largest African migrant population in Asia [15]. International migrants from developing countries, including Africans, are concentrated in Guangzhou’s Xiaobei district [1], a business hub for Chinese and international traders.

Health care in China is often fragmented and challenging to navigate [19]. Access to care can be difficult and expensive, even for Chinese citizens [19]. Though foreigners/international migrants legally residing in China can access almost any health care service from Chinese health care facilities, they are not eligible to participate in China’s national health insurance plan. Formally employed foreigners in some sectors may qualify for Chinese health insurance schemes [20, 21], but there are no similar systems that reach unemployed foreigners in China. As a result, health care costs are typically paid for out of pocket, and there are few resources available for foreigners who lack adequate financial resources. Furthermore, many health care facilities request a form of identification, such as a passport or visa, which illegal migrants may be afraid to or not able to provide.

### Recruitment

From April to July 2014, female Ugandan sex workers in Guangzhou were recruited through contacts with Ugandan community leaders. Women were approached by a female community-based organization representative or through a local male community-based organization representative. Both representatives had a history of implementing local programs for African sex workers in Guangzhou. Potential participants were greeted and told about the study purpose and study details. Appointments were then scheduled with women who were interested in participating according to women’s schedules. Participants were recruited until data saturation was reached. Participants were primarily recruited from the Xiaobei district, where many Africans in Guangzhou live. To compensate them for their time, participants were provided 100RMB (~$16 USD).

### Procedures

Participants were female, 18 years of age or older, had migrated from Uganda to Guangzhou, and reported having exchanged sex for money or gifts. Trafficking status was defined based on the criteria of the UN Trafficking in Persons (TIP) protocol, where a person is recruited by means of force, fraud, or abuse of power for the purpose of exploitation [22]. Most trafficked women in our study were deceived about job opportunities in China, had their travel organized and paid for by the trafficker, and then were forced to engage in sex work to repay their debts upon arrival in China. Participants were often forced through the use of physical violence or threats of violence and incarceration. Participants were classified as “legal” if they had a current valid passport and Chinese visa.

Each participant completed a face-to-face in-depth interview approximately an hour in length with one of two trained interviewers. One interviewer was an English-speaking African-American woman and the other was a student from Kenya studying at a medical school in the US. The shared racial background between interviewers and participants helped participants feel more comfortable and open during interviews. All interviews were conducted in English, with assistance from a Ugandan community leader in a few interviews where participant English skills were limited. In some interviews, the female community-based organization representative was also present. This was done to build trust in the research study and ensure that women felt comfortable during the interview. Interviews were conducted at the local hospital in a quiet, closed office or in a quiet nearby location that women trusted (e.g. the participant’s home or a shared community space).

An interview guide was designed to explore participants’ health-related experiences in Guangzhou and document barriers to health care access across 3 key domains: trafficking and legal status, economic disenfranchisement, and social isolation. The selection of these key domains and themes was informed by a literature review of existing research on barriers to health care for migrant sex workers, followed by field testing to capture emergent themes. The semi-structured interview guide was collaboratively developed by the research team during the literature review and adapted with input from several community members. Field test interviews were then performed with 7 local African women in the community (primarily with sex workers, but also with other African women in the community). The guide was further adapted based on their suggestions. The research team met to discuss the literature review and field test findings to develop a final interview guide. Interviews were semi-structured, allowing for flexibility depending on the participant’s comfort level around sensitive topics and exploration of emergent themes.

### Ethics approval and consent

No identifying information was collected on participants. All participants were told their answers were anonymous, would be kept confidential and only seen by approved research staff, and that they could choose to stop the interview at any time. All participants provided oral informed consent, and a form was signed by the interviewer to document consent. Institutional review boards at the Guangdong Provincial Center for Skin Diseases & STI Control and the University of North Carolina at Chapel Hill approved the study.

### Data analysis

Interview transcripts were analyzed using an *a priori* coding framework based on the 3 key domains and literature review, followed by open-coding to capture emergent themes [23]. The collaborative process of building the codebook and coding the data involved several steps [24]. First, a core group of 2 analysts read through the same sample of 5 interviews and independently generated an initial list of codes. The list of codes was shared with the rest of the research team. The codes were discussed and a codebook was constructed for the initial round of coding. All transcripts were coded by two independent researchers and compared in a coding conference to manage differences. Following the coding conference, data were resorted using the updated coding schema. Transcripts were compared to check reliability and consistency in the application of codes across transcripts. Coding responsibilities were divided among 2 analysts who met regularly to discuss the transcripts and refine the codebook as needed. This method involved multiple readings of transcripts and analytical induction via open and axial coding of data using NVIVO software (version 10, Doncaster, Australia) to thematically organize transcripts.

## Results

### Participants

In total, 19 Ugandan women participated in this study. Fifteen participants reported having between 1 and 5 children living back in Uganda. At the time of the interview, women had been living in Guangzhou from three months to 3 years, with a median of 15 months. Reported ages ranged from 22-40 years old. None of the participants had a valid visa, and 3 women had lost their passports in Guangzhou. Ten women reported sending remittances, or payments, home to support their children and families (See Table 1).

**Table 1:** Demographic characteristics of female African sex workers in Guangzhou, China, 2014, (*N* = 19)

*Characteristics*	*Number*
Age, *range (median)*	22−40 years (29 years)
Length of time in Guangzhou, *range (median)*	3−36 months (15 months)
Children, *n (%)*	
0	4 (21.1%)
1	5 (26.3%)
2	5 (26.3%)
3	2 (10.5%)
4	2 (10.5%)
5	1 (5.3%)
Had been trafficked to China, *n (%)*	12 (63.2%)
Did not have a valid Chinese visa, *n (%)*	19 (100.0%)
Reported sending remittances home, *n (%)*	10 (52.6%)
Reported losing passport in Guangzhou, *n (%)*	3 (15.8%)

### Trafficking and legal status

Twelve women reported a history of trafficking. These women reported not knowing they were coming to Guangzhou to engage in sex work, but thought that they were coming for another form of work, such as working in a clothing store or hair salon. After arriving in China, women were told there were no jobs for them, and they were forced to participate in sex work in order to repay their debts. One participant described the following:

*After the nursing course, I got this deal whereby somebody told me you can come this side, you can start working, there are jobs. It was somebody who brought me here, and then I started working in this prostitution, yeah. … So this person sends the money from here, for everything to be done the visa stuff, the ticket so that when you come this side, they just get like… a house, you’ll be staying here. Then they come take your passport, after finishing that money, owing that money which brought you here, then they give you back your passport. …. I didn’t know* [I would be prostituting] *because they told me there are shops, you can clean the floors, you can work, like in the salon.* – Female trafficking victim from Uganda, no children

By the time these women had repaid their debts to their traffickers, their visas had expired. They had no other source of income, means of legal employment, or way to return home. In order to return home, they were required to pay a $2,000 overstay fine (and a $300 fine for any children), a $300 registration fine, and the cost of their return ticket (and their child’s, if they had one) (A. Watson, personal communication). Women could not afford to pay for overstay fines and plane tickets home. These trafficked Ugandan women became stuck living illegally in China and forced to continue sex work as a means of survival.

The feeling of being stuck in China was shared by trafficked and non-trafficked women. Like the trafficked victims, non-trafficked women were unable to afford to return home and had overstayed their visas. Lack of documentation prevented them from getting jobs in the formal sector, contributing to continued sex work. The Ugandan women interviewed were highly afraid of being detained in a Chinese jail and were desperately struggling to save money to pay for their overstay fines and return tickets.

Ugandan sex workers inhabited a legally precarious situation both because of their engagement in sex work and because of their lack of a valid visa. Women were under constant fear of being arrested and concern about visas was expressed by each woman in our study. Women reported that visa checks were regularly performed by police on Tuesdays and Thursdays and that they did not leave their rooms during those days.

Lack of legal documentation was a major barrier to health services access at hospitals. Because many hospitals ask for identification (though not a valid visa specifically) and since all study participants were illegal migrants and did not possess a valid visa, many were afraid they would be arrested if they went to the hospital.

*When you go [to the hospital] they ask you for your identification and when you show them your passport it will be expired. Maybe you can be caught* [by the immigration authority or police] *like that.* – Female from Uganda, mother of 5 children

As a result of these fears, many women reported managing health care conditions on their own or seeing pharmacists in lieu of attending a hospital. Though most women believed that higher quality health care was available in hospitals than in other health care venues, such as pharmacies, they felt hospitals were out of reach for them because they lacked a valid visa.

*Big hospitals are better, they test you* [for] *everything. Because you go to* [a pharmacist], *she don't check. I showed her my anus she said, ‘Yeah this stuff is coming out,’ she say ‘Take this medicine.’ But she never took her time like a doctor… check what is the cause of it.* – Female trafficking victim from Uganda, mother of 3 children

Pharmacies were viewed as a low-barrier alternative to health care at hospitals or clinics. Women noted that pharmacists did not require identification or evidence of visas. There was also the perception among women in the sample that the pharmacist would treat several ailments and could distribute both traditional and modern medicine. One English-speaking pharmacist was well-known and trusted among sex workers.

*There is a woman in (street name). She’s called (nickname). She treat many prostitutes… I don't know which kind of doctor. I don't know which kind of sickness. But for her, she’s a doctor of everything. You understand?…It’s like a pharmacy. She work in herbal medicine and this medicine. All prostitutes are going there. All prostitutes in Xiaobei know (nickname).* – Female trafficking victim from Uganda, mother of one child

Thus, Ugandan sex workers chose the care that was most easily accessible to them, even though they believed hospital care to be higher quality.

### Economic disenfranchisement

Women’s impoverished status served as another primary barrier to health service access. Participants received relatively low wages from sex work (around $16 USD for one sexual act) and were being extorted by landlords who charged exorbitant prices for rent (up to $16 USD per day) because the women lacked valid documentation. Family economic need in their home countries compounded the financial position of these women. Half of the sample reported sending remittances home to support children and family members. Women reported a significant amount of pressure to financially support their families, and sending money home was prioritized over treating any non-life threatening illnesses.

*Each week* [my family gets money]*, because money of here has value… if you get 600* [RMB] *it is 100* [US] *dollar and in my country it is 250,000, so that is good money. So if I send them that amount they could buy things to eat, things to drink, and they could meet school dues. And getting 600 it means 6 men. If a night I can get three, then I pay my room then I eat a little then I keep. I keep like 200 or whatever… Right now* [my kids are] *at school, I have to send money for them. Their father doesn’t buy them—my neighbors call me “your kids are never at school. They’re at home.” So at least I get some money, what I have, I share with them, I send to them. They go to school.* – Female participant from Uganda, mother of five children

Largely due to financial concerns, seven women accessed medications from home via a friend who would come to Guangzhou. Medications identified included pain killers, malaria medication, HIV medication and antibiotics.

*Sometimes you don't have enough money. And some Ugandan women and Ugandan men bring medicine from Africa. So if you go there you tell them they can give you. But they are not doctor, they just buy medicine and bring. ….They bring small little* [amount]…*.Sometimes if it’s your friend you can pay small money or they can give you for free.* – Female trafficking victim from Uganda, mother of four children

Additionally, when going to the pharmacy, women’s choices were influenced by cost. They stated that the pharmacist would pull out several different medications, and they would choose the cheapest one.

*You keep on showing sign that the pain is here, then she understands you, so she brings out medicine. Some are expensive, some a little bit, then you choose that one that is not expensive. You say give me this.* – Female participant from Uganda, mother of 5 children

Lack of financial resources also appeared to be a barrier to HIV testing for some women. Women reported that they were afraid if they tested HIV positive, they would not be able to afford treatment. Such beliefs served as a disincentive for HIV testing.

*In China, you go to that hospital where they treat HIV, you have to be a resident because they don’t pay for that, so you have to pay. Even someone from Guangzhou cannot go to another place like Nanhai to get treatment. Someone from another city cannot get treatment from here, so you have to go back to where you were raised, then you can get treatment. So what about foreigners?* – Female participant from Uganda, mother of four children

Thus, insufficient financial resources impacted women’s health care access in multiple ways, including preventing acquisition of effective treatment and medication and discouraging women from accessing important sexual health screenings.

### Social isolation

Largely due to fear of discrimination as Africans and their trafficking and/or illegal migrant status, participants reported deep feelings of mistrust about Chinese government-affiliated organizations, including hospitals, and towards many Chinese individuals. Except for social networks with other sex workers, they described overall social isolation from society in general. Women perceived that they would be treated poorly or with disrespect from Chinese health professionals.

*Now in China they say* [it’s a problem for Africans] *to go to the hospital. You African, if they see you there they call police. They scared. They say that Chinese don’t want to touch African skin, you understand? They can call police; most of* [us] *are in overstay. Maybe they cannot treat* [us] *very well.* [African] *ladies don’t believe that Chinese can treat you very well.* – Female trafficking victim from Uganda, mother of one child

In addition to being discriminated against in China, women were also treated poorly by their friends in Uganda, due to the stigma attached to African women working in China.

*I try to call people to help me* [in Uganda]*. I didn’t know that it’s bad to* [be a] *woman in China. They know that in China are only prostitutes…I didn’t know it’s that bad. Everyone you try to ask for help, he ask you how you go there and you start to talk about yourself. They laugh, they abuse you. “Why you go there? You want to say that your mind is little? You didn’t know that China is only for prostitutes?” I get tired because everyone used to abuse me. I got a little bit disappointed…disappointment of myself to be ashamed in people this way.* – Female trafficking victim from Uganda, mother of one child

Thus, Ugandan sex workers were socially isolated from both their community of origin and Chinese society. Lack of adequate social support networks and fear of discrimination served as additional barriers to health care access.

## Discussion

Globalization and expanding Chinese-Ugandan trade relations have resulted in mass migration of men and women from Uganda to China. Though men often pursue jobs in business or trade, many Ugandan women in search of better economic opportunities are trafficked to China. Others move to China of their own accord to seek jobs in business or trade and end up becoming sex workers when they are unable to find legitimate employment. Our study examined barriers to health service access among trafficked and non-trafficked female Ugandan sex workers in Guangzhou, China. Previous research on sex workers in China has primarily focused on native Chinese sex workers [25, 26]. This study expands the existing literature by examining the inequalities Ugandan sex workers face in accessing health care in China. This study also expands the literature by illustrating some of the unintended consequences of increased Africa-China engagement.

Our study identified several barriers to health care access among female Ugandan sex workers in Guangzhou. Consistent with studies among sex workers in China [26], Asian countries [27], and other countries [28–30], lack of legal status and a valid visa were key barriers to health service access among women in our study. Fear of being arrested and the perception that hospitals required a valid visa to obtain health care services discouraged women in our study from accessing hospital services. Women felt safer accessing treatment from a trusted neighborhood pharmacy than from local hospitals. Women also accessed care from informal doctors and by receiving medication from incoming migrants. Accessing alternative sources of health care is common among individuals who cannot access the formal health care system [26, 28, 29]. However, by limiting health care access to these alternative sources of care, sex workers do not obtain needed services, such as HIV/STI testing and appropriate treatment by medical professionals. Although proof of identification is not legally required to obtain care at Chinese health care facilities, receptionists will often ask for a passport to record the patient’s name and personal information. A policy change that allows sex workers to access care without presenting formal identification could help facilitate African sex workers’ access to health services. However, informing African sex workers about such a change in policy may be difficult and, since many African sex workers’ mistrust Chinese health providers, they may still be afraid of going to a Chinese health care facility.

Similar to Chinese female sex workers [31, 32] and sex workers globally [33–35], poverty was also a major barrier to health service access among Ugandan sex workers in China. Women in this sample reported limited financial means in their home country, and remained impoverished in China. Since most women did not speak Chinese and could not be formally employed, they had few options to make money other than selling sex. Recent increases in the number of police officers in the Xiaobei district have restricted women’s movements even more (Ugandan consulate member, personal communication), making it more difficult for them to find clients and obtain sufficient financial resources to meet their basic needs, including being able to afford health care services. This is particularly problematic for diseases that necessitate early and continued treatment, such as HIV. For example, foreigners who are HIV positive are not able to legally access free antiretroviral therapy (ART) treatment. Although China has free ART pilot programs that cover foreign HIV-infected individuals, such as Burmese brides in Yunnan Province [36], they have not been widely implemented. Black market pricing makes ART treatment inaccessible to many. Further programs are needed for foreigners living with HIV who are not covered by existing policies and programs.

The majority of participants in our study were victims of human trafficking. Women who were trafficked had to pay several thousand dollars of debt to their traffickers before they were released. By the time they had paid their traffickers, their visas were already expired. Some women were able to find a sponsor who could pay for overstay fines and plane tickets, but still faced administrative and legal difficulties. Under the current system, Chinese immigration authorities will often place the woman in jail even if she can pay the overstay fines and plane tickets [13]. These financial and legal barriers made it impossible for women to return home and created a persistent feeling of being stuck in China. Changes in legal policies that would allow Ugandan women to return home without paying exorbitant overstay fines or spending an excessive amount of time in prison would enable these women to leave the sex industry in China and seek health care in their home country.

Trafficking policies in a range of settings limit victim access to services, but also suggest potential areas for reform to expand medical services. For example, trafficking policies in Canada are primarily focused on tight border control, visa restrictions and criminal prosecution, and are less focused on victim resources, such as social and legal assistance, protection, and health care [37]. However, unlike some other countries where trafficking victims are given refugee status or returned to their home countries, trafficking victims in China are often forced to remain in the country until they have paid their overstay fines [38]. Alternatively, they may choose to be detained in jail for an undisclosed length of time, often several months, as punishment for their overstay before being sent back to their home country [38]. Health policies less focused on punishing trafficking victims and more focused on providing medical services to trafficked victims could be useful. Local variation in implementation of Chinese laws [39, 40] provides sufficient flexibility for pilot medical programs that better appreciate the needs of trafficked sex workers in China. Experience from the HIV literature [41, 42] suggests that small pilot programs may help gradually shift local policies.

Our study has several limitations. First, this was an exploratory study among a small sample of female migrant Ugandan sex workers in Guangzhou, China. It is possible that our sample did not fully capture the diversity of experiences of this population. Just as the women we interviewed feared legal repercussions from seeking health services, some women may have been afraid to be interviewed by our research team. However, the fact that all of the women we interviewed were also illegal suggests that this was not a barrier to study participation. Second, we relied on self-reported experiences and behaviors provided by participants. Social desirability bias may have influenced women’s responses and made them hesitant to disclose sensitive information, such as information about their personal and sexual lives. However, women in our study did reveal much information about sensitive and difficult topics. The connection between our interviewers and African culture may have created a greater comfort level within the interview exchange and facilitated the rich narratives obtained.

## Conclusions

Our study reveals that female migrant Ugandan sex workers face additional barriers to health service access that are not faced by local Chinese sex workers. In particular, lack of a valid visa is a substantial barrier to access to health services. Not only are there misperceptions surrounding the need for a valid visa in a hospital, but lack of a visa also inhibits women’s movements, is a financial limitation, and prevents them from returning to their home countries. Given their high level of vulnerability and lack of resources, ensuring Ugandan sex workers have equitable access to effective and continuous health care in China is a challenge.

Our findings suggest policy and research implications. At the policy level, China has already implemented greater visa restrictions for Ugandans, thereby greatly decreasing the flow of trafficked Ugandan women into China [13]. Additional changes that allow remaining women to return to their native countries without paying overstay fines or serving jail time would enable these women to search for other jobs and connect to health services (and HIV treatment, if necessary) in their home countries. Furthermore, other options should be explored for expanding access to health care services, such as providing low-cost health care options or establishing pilot programs to include foreigners in domestic health plans. Such changes are urgently needed to enable female Ugandan sex workers to access necessary health care services. From a research perspective, we need to have better estimates of the total population of Africans and trafficked individuals in China and a more comprehensive understanding of their needs and challenges.

In conclusion, female African sex workers face many barriers to equal health care access in Guangzhou, China. Primary barriers to health care access are trafficking and legal status, economic disenfranchisement, and social isolation and discrimination. Policy changes and the implementation of programs that specifically target vulnerable African women in China are needed to ensure equitable access to health care for female migrant African sex workers.
